# Bile Leakage after Liver Transplantation Owing to Stricture of Afferent Jejunal Loop Caused by an Intussusception Valve after Biliary Atresia Surgery: A Case Report

**DOI:** 10.70352/scrj.cr.25-0209

**Published:** 2025-05-28

**Authors:** Hironobu Ito, Ryusuke Saito, Masaki Sato, Kyohei Kasuda, Naruhito Takido, Hiroyuki Ogasawara, Yoshihiro Shono, Muneyuki Matsumura, Ryuji Okubo, Kengo Sasaki, Atsushi Fujio, Hironori Kudo, Kazuaki Tokodai, Motoshi Wada, Michiaki Unno, Takashi Kamei

**Affiliations:** Department of Surgery, Tohoku University Graduate School of Medicine, Sendai, Miyagi, Japan

**Keywords:** intussusception anti-reflux valve, liver transplantation, biliary atresia, Kasai operation

## Abstract

**INTRODUCTION:**

Biliary atresia (BA) is a progressive cholangiopathy in neonates that results in biliary cirrhosis and liver failure without early intervention. Hepatic portoenterostomy (Kasai operation) remains the standard treatment, significantly improving survival rates. However, postoperative cholangitis is a major determinant of prognosis. To prevent cholangitis, various surgical modifications, including anti-reflux procedures such as intussusception anti-reflux valves (IAV), have been introduced. Although IAV has been widely adopted, some reports suggest that long-term survivors may develop afferent jejunal limb stenosis, leading to complications such as cholangitis and intestinal obstruction. Herein, we report a case of afferent jejunal loop stricture caused by IAV, which became symptomatic after liver transplantation (LT).

**CASE PRESENTATION:**

A 34-year-old man with a history of BA underwent Kasai operation with IAV and spur valve at 77 days of age. Despite experiencing recurrent cholangitis in adulthood, he survived with his native liver until developing liver cirrhosis and porto-pulmonary hypertension, necessitating deceased donor LT. Preoperative imaging revealed portal vein obstruction and dilated collateral circulation. During LT, severe adhesions and afferent limb dilation were observed, requiring a 30 cm resection of the jejunal limb. Postoperatively, he developed cholangitis, and imaging on postoperative day 16 revealed an anastomotic leak with an intra-abdominal abscess. Retrospectively, CT image before LT demonstrated the dilatation of the afferent limb and the stricture due to IAV was highly suspected. Double-balloon endoscopy confirmed complete afferent limb obstruction due to IAV-related stenosis. Surgical reconstruction with resection of the obstructed Roux-en-Y limb and creation of a new hepatojejunal anastomosis was performed. The patient recovered well and was discharged on postoperative day 45 without further complications.

**CONCLUSIONS:**

This case highlights the possibility for late-onset afferent jejunal stricture due to IAV in BA patients undergoing LT. The narrowing likely results from long-term fibrotic changes after 34 years from BA operation. Given the increasing number of BA survivors receiving LT, awareness of IAV-related complications is crucial. In cases with suspected afferent limb stenosis, preoperative assessment and consideration of jejunal limb resection during LT may help prevent postoperative complications.

## Abbreviations

BA
: biliary atresia
CT
: computed tomography
IAV
: intussusception anti-reflux valves
LT
: liver transplantation
POD
: postoperative day

## INTRODUCTION

BA is an idiopathic cholangiopathy that arises in newborns and leads to progressive inflammatory obstruction of the extrahepatic bile ducts. Without treatment, BA inevitably results in biliary cirrhosis and liver failure within the first or second year of life.^1)^ Hepatic portoenterostomy, commonly referred to as Kasai operation, is the essential surgical treatment that has significantly improved patient prognosis.^2,3)^ However, postoperative cholangitis significantly impacts the prognosis of patients with BA. Therefore, to prevent postoperative cholangitis, various surgical techniques have been implemented,^4)^ including external drainage procedures such as the Suruga II method.^5)^ Additionally, anti-reflux procedures, such as the double Roux-en-Y method and Roux-en-Y reconstruction with an anti-reflux intestinal valve, have been performed.^6–8)^ In the 1980s, hepatojejunostomy with an IAV was introduced to prevent cholangitis and was widely accepted.^9,10)^ On the other hand, there are some reports that IAV may cause stenosis of the afferent limb in the long-term survivors resulting in various complications such as strangulated intestinal obstruction or cholangitis due to cholestasis.^11,12)^ Herein, we report a case of afferent jejunal loop stricture caused by IAV after BA surgery, which became apparent post-LT.

## CASE PRESENTATION

**Fig. 1:**
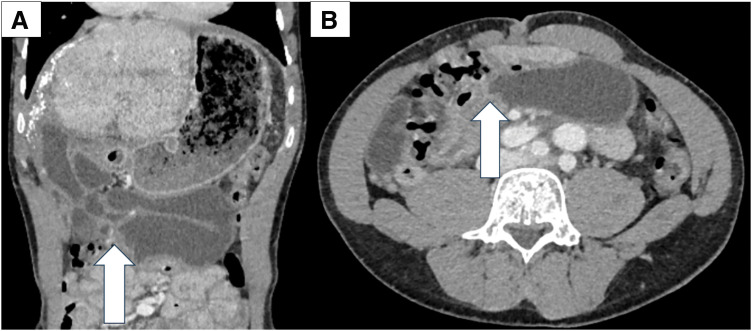
Preoperative CT image. (**A**) Dilatation of the afferent limb with beak sign was observed in coronal view. (**B**) Axial view. White arrow: Beak sign.

**Fig. 2:**
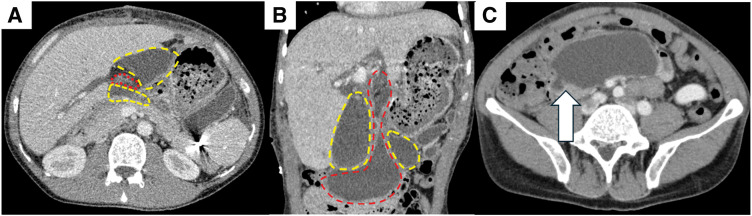
CT image on POD 16. (**A**) Abscess area surrounding the cholangiojejunostomy was observed by axial view. (**B**) Coronal view of the image. (**C**) Beak sign and dilatation of the proximal bowel by axial view. Red dot area: Dilated afferent jejunal loop, Yellow dot area: Abscess, White arrow: Beak sign.

**Fig. 3:**
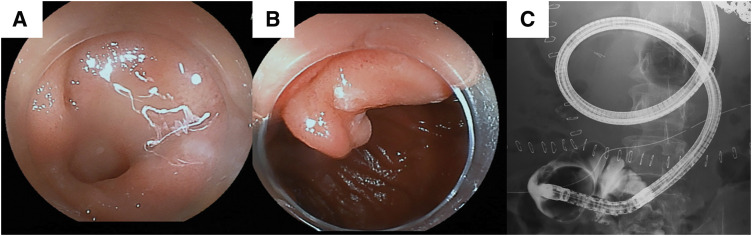
Double balloon endoscopy and retrograde cholangiography. (**A**), (**B**) Endoscopic examination revealed a valve-like structure, however, the lumen could not be observed. (**C**) Even with the use of a contrast agent, the jejunum loop was not visualized.

**Fig. 4:**
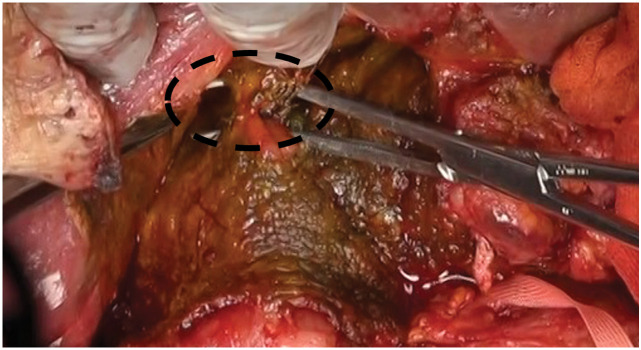
Picture of the second operation. The cholangiojejunal anastomosis was almost dehisced, with approximately half of the circumference disrupted. Black dot area: Cholangiojejunal anastomosis site.

**Fig. 5:**
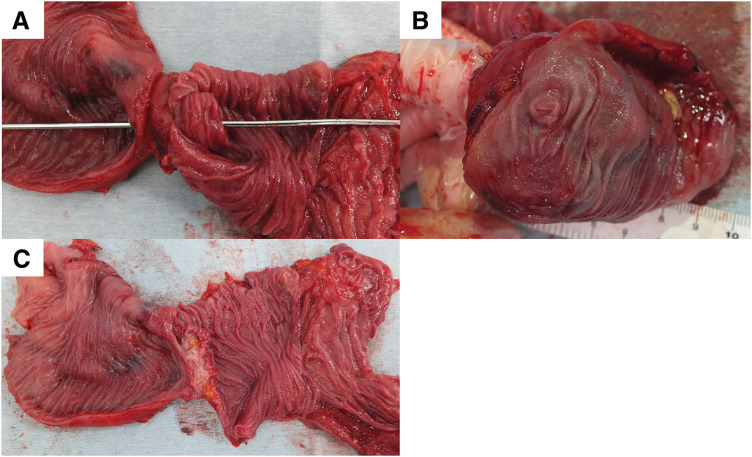
Resected afferent jejunal limb. (**A**)–(**C**) The lumen was almost occluded by the fibrous tissue, with a pinhole.

The patient was a 34-year-old man who underwent Kasai operation with IAV and spur valve for BA 77 days after birth. There was no major complication in the postoperative course in his childhood and he had experienced only 3 episodes of cholangitis. As the liver function got worse, some complications due to liver cirrhosis happened including intestinal bleeding in his thirties. He was diagnosed with porto-pulmonary hypertension 10 years ago, and prescribed 10 mg of Tadarafil and 25 mg of Eplerenone per day. As the diagnosis was liver cirrhosis after BA with porto-pulmonary hypertension, deceased donor LT was performed when he was 34-year-old (Model for end-stage liver disease score: 12). Preoperative enhanced CT showed an atrophic liver and obstruction of the portal vein with dilated collateral circulation. During the operation, there were strong adhesions especially around the liver hilum. We could observe the dilatation of the afferent limb in the operation. Due to adhesion, we cut 30 cm of afferent jejunum, including hepaticojejunostomy. The superior mesenteric vein of the recipient was anastomosed with the portal vein of the donor using jump graft (iliac vein). Choledocho-jejunostomy with drainage tube was also performed. Although the initial postoperative course was stable, on POD 6, the patient developed a fever exceeding 38°C, accompanied by an elevation of the inflammatory markers and hepatobiliary enzymes. Given the suspicion of cholangitis, empirical antibiotic therapy was initiated. Despite the continued antibiotic treatment, cholangitis symptoms fluctuated and persisted for over a week. Retrospectively, the dilated bowel loop in the upper abdomen was considered to be the afferent limb with a beak sign (**Fig. 1**). CT image on POD 16 identified an intra-abdominal abscess surrounding the hepatojejunal anastomosis (**Fig. 2**). Consequently, percutaneous abscess drainage was performed, yielding a bile-like fluid, suggesting an anastomotic disruption secondary to jejunal limb stricture. To address this complication, double-balloon endoscopic retrograde cholangiography was performed to assess and attempt dilation of the afferent limb. However, complete luminal obstruction was observed (**Fig. 3**). The obstruction was attributed to an anti-reflux valve constructed during the previous Kasai operation, necessitating surgical reconstruction of the biliary drainage route on POD 25. The previous anastomosis of biliary tract was almost dehisced, with approximately half of the circumference disrupted (**Fig. 4**). The obstructed Roux-en-Y limb was resected, and a new limb was created, followed by reconstruction of the hepatojejunal anastomosis with the biliary drainage tube placement. On examination of the resected intestine, its lumen was nearly occluded by thickened fibrous tissue, with a pinhole (**Fig. 5**). Postoperatively, the patient exhibited rapid resolution of cholangitis, with no evidence of biliary leakage. He was discharged on POD 45 with stable laboratory findings.

## DISCUSSION

Postoperative cholangitis has been reported to occur in approximately 40%–80% of patients with BA following Kasai operation, significantly contributing to poor prognosis.^13–15)^ Therefore, various anti-reflux procedures including intestinal anti-reflux valve have been reported.^4,9,11)^ There are 2 main types of anti-reflux valves: IAV and spur valve. The most significant difference between the spur valve and the IAV is that the spur valve is approximately semi-circumferential, whereas IAV is fully circumferential and is more likely to cause stenosis of the afferent limb. This is the first report that the afferent jejunal loop stricture caused by IAV became apparent due to bile leakage just after LT.

Since 1980, when Tanaka et al. first reported the addition of an anti-reflux valve,^9)^ several retrospective studies have suggested that anti-reflux valves reduce the incidence of postoperative cholangitis compared with the standard Kasai operation.^8,10,16–18)^ However, Ogasawara et al. conducted the first prospective study in 2003,^19)^ comparing 10 patients with IAV to 11 patients without it to evaluate its efficacy in preventing cholangitis. The study found no significant difference in the occurrence rate of cholangitis between the two groups. However, due to the small sample size, the possibility remains that the study lacked sufficient power to detect a true difference. Through those decades of discussions, the effectiveness of anti-reflux valves remains a subject of debate.

According to the National Registry of BA in 2022 in our country, the most common procedure is the simple long Roux-en-Y (71.0%), followed by Roux-en-Y with anti-reflux valve (14.5%).^20)^ There is a report from China that the spur valve construction was performed in more than half of the centers (51%).^21)^ In our department, Roux-en-Y anastomosis with double valve (IAV and spur valve) was the standard procedure in 1992–2000. However, Roux-en-Y anastomosis with spur valve is the standard procedure from 2000 to date. One of the reasons for the change in procedure during this period was the suboptimal rate of bilirubin reduction, possibly due to impaired intestinal motility caused by the double valve. There are no studies comparing the efficacy of spur valves and IAV by valve types; however, the spur valve is currently considered as the mainstream option.

There are some reports that state IAV caused some postoperative complications such as repeated cholangitis or strangulated intestinal obstruction, as summarized in **Table 1**.^11,12,17,22)^ According to these reports, these complications occurred in the long-term survivor with IAV, except for case 4. These complications are likely to result from stenosis that develops over a long-term course, occurring at least 15 years after the first operation, and subsequently leading to various clinical manifestations. We could not find a report that spur valve caused the stenosis of afferent limb or ascending cholangitis. In practice, it is extremely difficult to distinguish whether cholangitis and liver failure are caused by the valve, the progression of the underlying disease, or a combination of both. Even in LT cases after Kasai operation, cases where the valve played a harmful role are likely overlooked. In our case, he was diagnosed with porto-pulmonary hypertension 10 years before and the dilatation of the afferent limb was first observed by CT 1 year before LT. Therefore, it remains unclear to what extent IAV was involved in the development of liver cirrhosis or porto-pulmonary hypertension.

**Table 1:** Complication associated with anti-reflux valve after Kasai operation

Case (Ref.)	Age	Sex	Valve type (Spur or IAV)	Period after Kasai operation	Complication
1 (11)	24	F	IAV	24 years	Torsion of afferent limb (strangulated ileus)
2 (12)	15	F	IAV	15 years	Cholangitis from cholestasis
3 (12)	26	M	IAV	26 years	Cholangitis from cholestasis
4 (17)	2 months	M	IAV	5 days	Jejunal perforation
5 (22)	20	F	IAV	20 years	Intestinal obstruction caused by gallstone
Our case	34	M	IAV and Spur valve	34 years	Disruption of hepatojejunal anastomosis

F, female; IAV, intussusception anti-reflux valve; M, male

We could observe the dilatation of the afferent limb by CT before operation (**Fig. 1**). There are 3 possible hypotheses as to why narrowing of the afferent limb became apparent after LT. First, a large amount of bile was produced by the healthy liver after transplantation. Second, the intraoperative resection of 30 cm of jejunum reduced the reservoir capacity. Third, postoperative edema markedly exacerbated the existing stenotic region. It has been reported that the number of patients who survive with their native liver after Kasai operation is 44.5% at 20 years and 40.8% at 30 years.^20)^ Patients with IAV treated in 1980–2000 may require LT due to liver failure. Therefore, a similar case we reported this time could occur. To prevent postoperative complication after LT caused by the anti-reflux valve, we should check the operative procedure for BA for whether the anti-reflux valve was constructed or not, and additionally, the need to identify the type of valve. Hepatobiliary scintigraphy could have been one of the options for detecting stasis in the afferent limb.^23)^ In addition, resection of dilated afferent jejunum and reconstruction of Roux-en-Y anastomosis should be considered for cases with the dilatation of the afferent limb preoperatively.

## CONCLUSIONS

We report a case of afferent jejunal loop stricture caused by IAV after BA surgery, which became apparent after LT. Stricture due to IAV can occur over a long-term course. In cases with intussusception valves or suspected stenosis of the afferent jejunum, reconstruction of Roux-en-Y anastomosis should be considered during LT.

## DECLARATIONS

### Funding

No funding was received for this report.

### Authors’ contributions

H. Ito and R.S. participated in all aspects of this study, including patient management, report conceptualization, and draft writing.

M.S., K.K., N.T., H.O., Y.S., M.M., K.S., and A.F.; managed the patients.

R.O., H.K., K.T., M.W., M.U., and T.K.; supervised the article.

All authors have read and approved the manuscript and agree to be held accountable for all aspects of this report.

### Availability of data and materials

The datasets supporting the conclusions of this study are included in this article.

### Ethics approval and consent to participate

This work does not require ethical considerations or approval. Informed consent to participate in this study was obtained from the patient.

### Consent for publication

Consent was obtained from the patient for the publication of this case report.

### Competing interests

All authors declare no competing interests for this article.
